# *Sida gathwalarum* (Malvaceae), a new species from India

**DOI:** 10.3897/phytokeys.274.191644

**Published:** 2026-05-18

**Authors:** Sumit Malik, Inam Malik, Vijai Malik

**Affiliations:** 1 Department of Botany, Chaudhary Charan Singh University, Meerut-250004, Uttar Pradesh, India Department of Botany, Chaudhary Charan Singh University Meerut India; 2 Patanjali Research Institute, Haridwar-249404, Uttarakhand, India Patanjali Research Institute Haridwar India

**Keywords:** India, Malvaceae, Meerut, mericarp, *Sida acuta*, *Sida angustifolia*, *Sida gathwalarum*, Uttar Pradesh

## Abstract

*Sida gathwalarum* (Malvaceae), a new species from Meerut, Uttar Pradesh, India, is described and illustrated based on morphological and phylogenetic evidence. Morphologically, the new species is similar to *Sida angustifolia* Mill. but consistently differs from it in leaf shape, varying from narrowly lanceolate to ovate; base cuneate to rounded; petioles without spiny emergence (spur) at the base; stipules linear-lanceolate and dissimilar in shape; and yellow styles of different sizes. The species is further characterized by indehiscent fruits with the fruiting calyx soft and compressible, the corolla retuse to nearly entire at the apex, and 5(–6) markedly heteromorphic mericarps that are trigonous–globose, coarsely rugose–tuberculate, and bear a shallow U-shaped apical notch. The seeds are subtrigonous, asymmetrical, and uniformly dull brown to grayish brown. These stable diagnostic traits from different populations support its recognition as a new species, *Sida gathwalarum* Sumit Malik, Inam Malik & Vijai Malik, **sp. nov**. Molecular phylogenetic analysis of nuclear ribosomal DNA (internal transcribed spacer) sequence data of 14 *Sida* species supports the phylogenetic position of the new species, *Sida gathwalarum*, within sect. *Sidae*. The results of molecular phylogenetic analysis show that *Sida gathwalarum* is closely related to *S. acuta*.

## Introduction

The genus *Sida* L. was established by Linnaeus with 10 species in Species Plantarum (Linnaeus 1753). The species of this genus are widely distributed across pantropical and subtropical regions (Mabberley 2017) and represent one of the most taxonomically challenging genera in tribe Malveae due to pronounced morphological plasticity. The genus comprises ca. 275 species worldwide (POWO 2025) and 21 species in India, including 17 species, two subspecies, and two varieties. Widely distributed Indian species include *S. acuta*, *S. cordata*, *S. cordifolia*, *S. spinosa*, and *S. rhombifolia*, whereas species such as *S. elongata*, *S. javensis*, *S. linifolia*, *S. pradeepiana*, *S. ravii*, *S. repens*, *S. subcordata*, and *S. schimperiana* are largely restricted to central and southern India (Pramanick et al. 2020). During recent field surveys (2023–2025) in North India, the authors observed several populations of an interesting species of the genus *Sida* from roadsides and wasteland in Meerut and Saharanpur, Uttar Pradesh, and Roorkee, Haridwar (Uttarakhand), India. A total of five samples of this new taxon were observed from the aforementioned localities (Fig. 4). On critical examination of the protologues (Linnaeus 1753; Miller 1768; Burman 1768) and type specimens (BM000603894, G00360092, LINN 866.1, 866.2, 866.3, 866.7, 866.12; K000659370; C10003059; MA656279; K000659357), it was evident that the material did not correspond to any known species of the genus *Sida*. A thorough review of the pertinent literature, namely Hooker (1872), Duthie (1903–1929), Kanjilal (1928), Clement (1957), Van Borssum Waalkes (1966), Babu (1977), Bhandari (1977), Fryxell (1985, 1988), Sharma and Sanjappa (1993), Sivarajan and Pradeep (1996), Sivadasan and Anil Kumar (1996), Tambde et al. (2016, 2020), Gavade et al. (2020), and Santhosh Kumar et al. (2001, 2021), together with expert comments on the identity of the specimens, further confirmed that the collected specimens could not be assigned to any described taxa. Consequently, the material is here described as *Sida gathwalarum* sp. nov.

## Materials and methods

### Morphological analysis

This study is based on examination of living specimens, herbarium specimens (DD and BSD), and high-resolution images of specimens housed at virtual herbaria (CAL, BSA, K, and P). Morphological observations were made on fresh and dried specimens collected from several localities (Fig. 4) of North India during 2023–2025. All measurements were made exclusively on living material. Eleven taxa representing nine species, one subspecies, and one variety were critically examined and compared. Specimens were studied under a Euromex DZ5040 microscope. Photographs were taken using Nikon D5600 and Canon EOS 1300D cameras. Among the collected specimens, certain populations initially appeared to represent morphological variants of *Sida angustifolia* Mill. (Miller 1768). Morphological terminology was adopted in accordance with Beentje (2012) and Simpson (2019). However, detailed morphological assessment revealed that these collections exhibited stable and consistent differences from *S. angustifolia* and did not correspond to any described taxa. This led to the recognition of a new species. Voucher specimens have been deposited in BSD and DD herbaria.

### DNA isolation, amplification, and sequencing

Genomic DNA was extracted from fresh plant material using the QIAGEN DNeasy Ultra Clean Microbial Kit (Barcode Biosciences, Bangalore, India; Cat. No. 12224-50) following the manufacturer’s protocol. The nuclear ribosomal internal transcribed spacer (ITS) region was amplified using specific forward and reverse primers (Chen et al. 2010; Cheng et al. 2016). Polymerase chain reaction (PCR) products were purified using the QIAGEN QIAquick PCR Purification Kit (Cat. No. 28104) to remove residual primers and nucleotides. DNA sequencing was carried out following the Sanger dideoxy chain termination method.

### Phylogenetic analysis

Chromatograms (in .ab1 format) obtained from Sanger sequencing were visualized and manually inspected using FinchTV v1.4.0 (FinchTV 2012). Low-quality and ambiguous base calls were trimmed to remove unreliable regions. High-quality forward and reverse sequences were assembled into consensus sequences using DNA Baser v5.21 (DNA Baser 2019).

A total of 18 sequences were included in the phylogenetic analysis. Of these, two sequences representing the new species, *Sida gathwalarum*, were generated by the authors from specimens collected at different locations. Additionally, two sequences of distinct species were retrieved from NCBI and used as outgroups. The dataset further comprised 14 sequences of different *Sida* species, of which 12 were obtained from NCBI, while two sequences (*Sida angustifolia* and *Sida spinosa*) were newly generated in this study (Table 2). The newly generated sequences will be submitted to NCBI following publication. Multiple sequence alignment was performed using the MAFFT v7 online server (https://mafft.cbrc.jp/alignment/server/) (Katoh and Standley 2013) with default parameters. The aligned sequences were downloaded in FASTA format and subsequently inspected and manually adjusted in MEGA v12.0.10 to eliminate poorly aligned and ambiguous regions (block trimming), ensuring positional homology (Tamura et al. 2021). The best-fit nucleotide substitution model was selected using jModelTest v2.1.10 based on the Akaike Information Criterion (AIC) (Darriba et al. 2012). The Kimura 2-parameter (K2) model was identified as the most appropriate and was applied to the dataset. Maximum likelihood (ML) analysis was conducted in MEGA v12.0.10 with 1,000 bootstrap replicates. Bootstrap support (BP) values were interpreted as 50–70% (low), 71–84% (moderate), and 85–100% (strong). Bayesian inference (BI) analysis was performed using MrBayes v3.2.7a (Ronquist et al. 2012) under the K2 model. Two independent runs were conducted with four Markov chain Monte Carlo (MCMC) chains for 1,000,000 generations, sampling every 100 generations. The first 25% of sampled trees were discarded as burn-in, and the remaining trees were used to generate a majority-rule consensus tree. Posterior probabilities were used to assess clade support. The Newick format tree prepared in MEGA was visualized in iTOL (https://itol.embl.de/).

## Taxonomic treatment

### Sida gathwalarum

Taxon classification

Plantae

DiplostracaMalvaceae

Sumit Malik, Inam Malik & Vijai Malik sp. nov.

E1DCDB70-37D6-5D97-B7AC-63436370BDC1

urn:lsid:ipni.org:names:77380111-1

Fig. 1

#### Type.

India • Uttar Pradesh: Meerut Cantonment, 29.007637, 77.714272, 16 November 2025, *Sumit Malik & Vijai Malik 1706* (Holotype BSD137791!; Isotypes BSD137790!; DD S-1012!, S-1013! & S-1014!).

**Figure 1.**
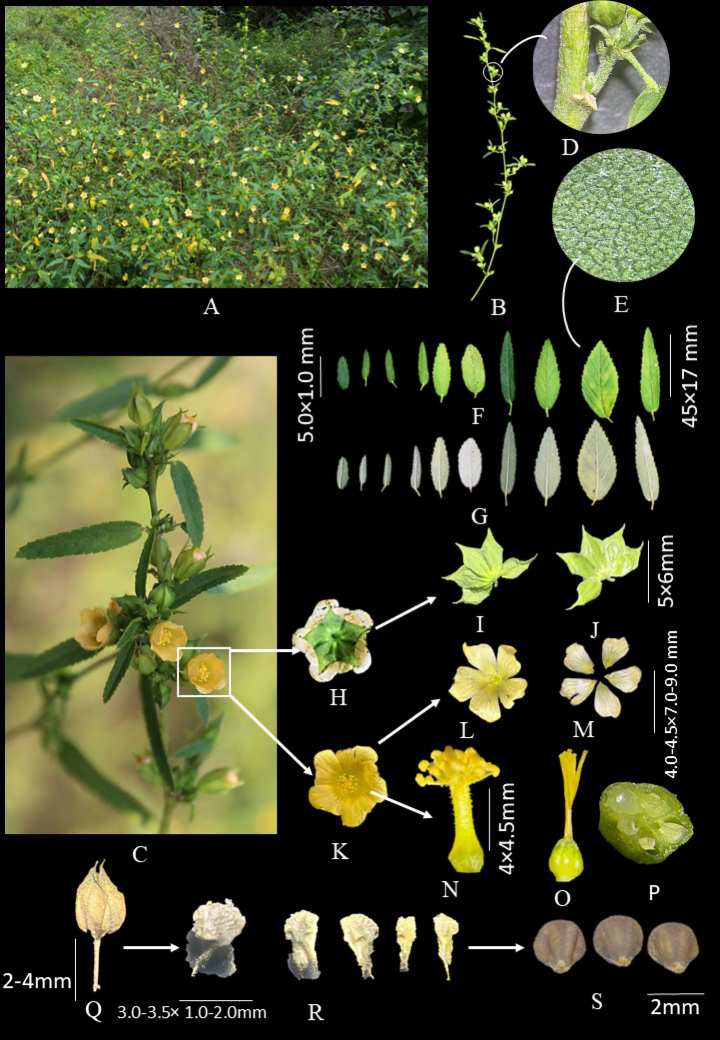
**A**. Habitat; **B**, **C**. Habit; **D**. Stem, stipules, and petiole; **E**. Abaxial surface of leaf showing stellate hairs; **F**. Adaxial surface of leaf; **G**. Abaxial surface of leaf; **H**. Lower surface of flower; **I**. Outer surface of calyx; **J**. Inner surface of calyx; **K**. Upper surface of flower; **L**, **M**. Corolla; **N**. Staminal tube; **O**. Style and ovary; **P**. T.S. of ovary; **Q**. Fruit with accrescent calyx; **R**. Mericarps; **S**. Seeds.

#### Diagnosis.

This species closely resembles *Sida angustifolia* Mill. in having, erect branched subshrubs, with ***branches*** alternate and spiral terete, pubescent with stellate hairs, calyx similar in shape with similar type of simple and stellate hairs in both species. Filaments and staminal tube also bearing similar type of simple and transparent glandular hairs but differing conspicuously in several vegetative and reproductive characters. Erect, up to 2 m tall (vs. 1 m tall). ***Leaves*** alternate and spiral, variable from narrowly lanceolate to ovate (vs. linear spear-shaped), base cuneate to rounded (vs. truncate or cordate at base), 3(-5) nerved at base (vs. only three nerves at base). Petioles 2.0–5.0 mm (vs. 5.0–45 mm) without spiny emergence (spur) at base (vs. three spiny emergences spur present at base of leaf). ***Stipules*** are linear lanceolate in shape, 1.5–3.0 × 0.5–1.0 mm, subequal (vs. 5.0–7.0 × 1.0 mm, falcate, equal in shape) furnished with both simple and stellate hairs along the margins and on both surfaces. Peduncle 1.0–2.0 mm; pedicel 1.5–2.5 mm, articulation usually present below the middle (vs. peduncles 2.5–3.0 mm; pedicels 1.0–2.0 mm in flowers, articulation above the middle), ***Petals*** retuse to nearly entire at apex (vs. deeply emarginate notch at apex). Styles dissimilar in size (vs. similar in size), yellow in color. ***Esquizocarp*** indehiscence (vs. dehiscence), fruiting calyx soft and compressible. ***Mericarps*** 5–6, heteromorphic (vs. mericarps five, not as in *Sida gathwalarum*), mature mericarps trigonous–globose, 3.0–3.5 × 1.0–2.0 mm (vs. 4.0–4.2 × 2.0–2.2 mm), their surfaces coarsely rugose to tuberculate, accrescent calyx hardly becomes empty, the mericarps remain enclosed and are not shed easily. Each mature mericarp possesses a shallow, broad, U-shaped apical notch. ***Seeds*** subtrigonous (vs. trigonous), slightly asymmetrical with unequal sides, one angle broader and more obtuse, dorsal ridge less sharp and more rounded, glabrous but shortly hairy at the hilum (vs. dorsal ridge very sharp, pointed, glabrous, granular at hilum), and uniformly dull brown to grey-brown (vs. variable in color brownish to black) (Table 1).

**Table 1.** Morphological comparisons of *Sida gathwalarum*, *Sida angustifolia*, and *Sida acuta* based on published literature and live specimens (Fig. 2).

Characters		*Sida gathwalarum*	*Sida angustifolia*	*Sida acuta*
**Plant**	**Height**	Up to 2 m tall	Up to 1 m tall (Gavade et al. 2020)	Up to 1 m tall (Sivarajan and Pradeep 1996)
	**Habitat**	Roadsides and wastelands	Roadsides and wastelands	Roadsides and wastelands
**Branches and leaf arrangement**		Alternate and spiral	Alternate and spiral	Distichous (Sivarajan and Pradeep 1996)
**Leaves**	**Shape and size**	Narrowly lanceolate to ovate, 5.0–45 × 1.0–17 mm	Linear spear-shaped (Miller 1768), 10–50 × 3.0–15 mm	Lanceolate to ovate, oblong or liner (Van Borssum Waalkes 1966)
	**Leaf base** (**Fig. 2ii)**	Cuneate to rounded	Truncate or cordate at base (Gavade et al. 2020)	Truncate (Sivarajan and Pradeep 1996)
	**Nerves at base**	3(-5) nerved	Only three-nerved (Gavade et al. 2020)	Three nerved at base (Sivarajan and Pradeep 1996)
	**Petiole length**	2.0–5.0 mm	5.0–45 mm (Gavade et al. 2020)	3.0–6.0 mm (Van Borssum Waalkes 1966)
	**Spiny emergences (spurs) (Fig. 2iii)**	Absent	Three spiny emergences (spurs) at base (Gavade et al. 2020)	Absent
	**Leaf indumentum (adaxial)**	Sparsely to coarsely pubescent (stellate + simple hairs)	Densely stellate tomentose (Gavade et al. 2020)	Hairy or glabrous (Sivarajan and Pradeep 1996)
	**Leaf indumentum (abaxial)**	Stellate hairs present	Sparsely pubescent above (Gavade et al. 2020)	Hairy or glabrous (Sivarajan and Pradeep 1996)
**Stipules**	**Size and shape**	1.5–3.0 × 0.5–1.0 mm, subequal, narrowly lanceolate to linear-lanceolate	5.0–7.0 × 1.0 mm, equal, ensiform, falcate (Gavade et al. 2020)	6.0–10 × 1.0–1.5 mm, one lanceolate or falcate other linear to filiform (Sivarajan and Pradeep 1996)
	**Stipule indumentum**	Simple + stellate hairs on both surfaces and margin	Densely pubescent with stellate hairs (in live specimen)	Simple hairs only on margin
**Flowers**	**Position**	Axillary, two or more	Solitary axillary (Gavade et al. 2020)	Axillary, solitary, by development of an accessory bud in clusters of 2–8 flowers (Van Borssum Waalkes 1966)
	**Petal size**	7.0–9.0 × 4.0–4.5 mm	6.0–6.5 × 3.0–4.0 mm (Gavade et al. 2020)	7–10 mm (Sivarajan and Pradeep 1996)
	**Petal apex (Fig. 2i)**	Retuse to nearly entire apex	Slightly falcate, blunt apex (Gavade et al. 2020)	Obliquely obovate, usually emarginate (Van Borssum Waalkes 1966)
	**Peduncle + pedicel**	Peduncle 1.0–2.0 mm; pedicel 1.5–2.5 mm; pubescent; longer or shorter than petiole	Peduncle 2.5–3.0 mm; pedicel 1.0–2.0 mm; slightly pubescent; shorter than petiole (Gavade et al. 2020)	Peduncle + pedicel 3–10 mm (Sivarajan and Pradeep 1996)
	**Articulation (peduncle-pedicel) (Fig. 2iv)**	Below middle	Above middle	Usually jointed ± at the middle (Van Borssum Waalkes 1966)
	**Calyx indumentum**	Outer surface pubescent (simple + stellate), inner glabrous	Densely stellate hairs present on outer surface, inner glabrous (in live specimen)	Outer surface pubescent or glabrous
	**Staminal tube**	ca. 3.5 mm, pubescent (simple + glandular hairs)	2.0–2.5 cm, stellate-pubescent (Gavade et al. 2020)	2–2.5 mm long glabrous or minutely pubescent (Sivarajan and Pradeep 1996)
	**Style size**	Unequal in size (Fig. 1O)	—	Equal style larger than filament
	**Style color**	Yellow	Yellow (Gavade et al. 2020)	White (in live specimen), pale yellow (Sivarajan and Pradeep 1996)
**Fruit (general)**		Indehiscent; calyx soft and compressible	Dehiscent; calyx firm and non-compressible	Dehiscent; calyx firm and non-compressible
**Mericarps (Fig. 2v)**	**Number and nature**	5(-6), heteromorphic	Five, homomorphic (Gavade et al. 2020)	6–10, homomorphic (Van Borssum Waalkes 1966)
	**Size**	3.0–3.5 × 1.0–2.0 mm	4.0–4.2 × 2.0–2.2 mm (Gavade et al. 2020)	2.0–2.5 mm long (Van Borssum Waalkes 1966)
	**Shape (mature)**	Trigonous-globose	Trigonous (Gavade et al. 2020)	Trigonous with acute angle (Sivarajan and Pradeep 1996)
	**Surface**	Coarsely rugose -tuberculate	Reticulate or wrinkled (Gavade et al. 2020)	Reticulate on the side below, reticulate or transversely rugose on the back (Sivarajan and Pradeep 1996)
	**Awns**	Apically two awns, 1.0–1.5 mm, simple and stellate hairy, divergent/parallel	Two awns ca. 2 mm, divergent covered with simple and stellate hairs (Gavade et al. 2020)	Two awns 1–1.5 mm long, divergent glabrous (Van Borssum Waalkes 1966; Sivarajan and Pradeep 1996)
	**Mericarp separation**	One mericarp separates easily; others remain attached to calyx cup	All mericarps separate easily	All mericarps separate easily
	**Notch between awns**	Shallow, broad, U-shaped	Notched between awn and lower portion of mericarp (Gavade et al. 2020)	Deep, narrow sinus
**Seeds (Fig. 2vi)**	**General**	Seeds are of different shape and size	All seeds are of same shape and size	All seeds are of same shape and size
	**Seed size**	Mature seed 2 mm long	0.18–0.18 mm long (Gavade et al. 2020)	2 mm long (Van Borssum Waalkes 1966)
	**Seed shape**	Subtrigonous, slightly asymmetrical	Ovoid, rounded trigonous (Tambde 2023)	Trigonous with rounded angles (Sivarajan and Pradeep 1996)
	**Seed surface (hairs)**	Glabrous, shortly hairy at hilum	Glabrous, granular at hilum (Gavade et al. 2020)	Glabrous, shortly hairy at hilum (Van Borssum Waalkes 1966)
	**Dorsal ridge**	Less sharp, rounded	Very sharp, pointed	Very prominent, thick
	**Apex**	Broadly rounded	Pointed	Slightly pointed
	**Seed width symmetry**	Asymmetrical	Symmetrical	Symmetrical
	**Color**	Dull brown to grey-brown	Brownish to black (Gavade et al. 2020)	More variable in color as observed in live specimen

**Table 2.** Species information for phylogenetic analyses and GenBank accession numbers.

Species name	Accession number
*Sida acuta* Burm.f.	KJ636990.1
	KJ636992.1
*Sida angustifolia* Mill.	1713
*Sida cordata* (Burm.f.) Borss. Waalk	JN542432.1
*Sida cordifolia* L.	JN542431.1
*Sida gathwalarum* sp. nov.	1706, 1707
*Sida rhombifolia* L.	MH768243.1
*Sida alnifolia* L.	KM073065.1
*Sida rhombifolia* subsp. alnifolia (L.) Ugbor.	MG596790.1
*Sida ravii* Sivad. & Anil Kumar	PV754328.1
*Sida repens* Dombey ex Cav.	OM752104.1
*Sida mysorensis* Wight & Arn.	KM073066.1
	OQ165159.1
*Sida spinosa* L.	1710
*Sida sivarajanii* Tambde, Sardesai & A.K. Pandey	MK829605.1
*Abutilon indicum* (L.) Sweet	KP092989.1
*Abutilon abutiloides* (Jacq.) Garcke ex Hochr.	KT966960.1

#### Description.

Erect, highly branched, suffrutex up to 2 m tall. Generally, stem green rarely bicolorous with one face green and the opposite brownish. Branches alternate and spiral, terete, pubescent with stellate hairs. ***Leaves*** 5.0–45 × 1.0–17 mm, alternate and spiral (Fig. 1B, C), variable from narrowly lanceolate to ovate, apex acute to acuminate, base cuneate to rounded, pulvinus, whole margin serrulate (Fig. 1F, G), adaxial surface sparsely to coarsely pubescent with minute stellate and simple hairs, abaxial surface densely covered with minute stellate hairs (Fig. 1E); venation pinnate, midrib prominent on lower surface, 3(-5)-nerved at base, lateral nerves 4–7 pairs. Petiole 2.0–5.0 mm with simple and stellate hairs without spiny emergence (spur) at base. ***Stipules*** narrowly lanceolate to linear lanceolate, 1.5–3.0 × 0.5–1.0 mm, subequal, 3–5 nerved, deciduous with simple and stellate hairs on margins and both surfaces (Fig. 1D). ***Flowers*** axillary, usually two or more, pale yellow, bisexual. Peduncle 1.0–2.0 mm; pedicel 1.5–2.5 mm, articulation usually present below the middle, longer or shorter than stipules. ***Calyx*** five-lobed divided in middle, lobes 5.0–6.0 × 2.0–3.0 mm, accrescent with fruits, campanulate, venation 10 nerved, margin ciliate, outer surface pubescent with simple and stellate hairs, inner surface glabrous (Fig. 1H–J). ***Corolla*** pale yellow, petals 5, 7.0–9.0 × 4.0–4.5 mm, retuse to nearly entire, twisted aestivation, minute simple and glandular hairs on outer surface and margin, 9–10 nerves from base, branched (Fig. 1K–M). ***Stamens*** numerous (27–30 in five bundles), monadelphous, staminal tube ca. 3.5 mm, pubescent with transparent simple and glandular hairs (vs. 2.0–2.5 mm long, stellate-pubescent), anthers yellow, reniform, filaments 1–1.5 mm, pubescent (same as on stamina tube). ***Carpels*** 5–6, ovary hairy, styles 3.8–4.5 mm, unequal in length, divided almost from the base, yellow, appressed hairs on style, stigma capitates, and yellow (Fig. 1N, O); axile placentation and ovules do not appear exactly equal in size (Fig. 1P). ***Esquizocarp*** indehiscence, mericarps 5(-6), heteromorphic (Fig. 1R), unequally filling accrescent calyx, fruiting calyx soft and compressible, accrescent calyx hardly becomes empty, the mericarps remain enclosed and are not shed easily (Fig. 1Q), mature mericarp trigonous–globose, 3.0–3.5 × 1.0–2.0 mm, upper side brown and lower side light brown, surface coarsely rugose–tuberculate with irregular lumps and wrinkles, not arranged in regular folds, one mericarp comes out and rest mericarps remain embedded within accrescent calyx, apically two awns, 1.0–1.5 mm, simple and stellate hairy, divergent or parallel, notch shallow, broad, U-shaped. ***Seed*** 2 mm long, subtrigonous, slightly asymmetrical, with unequal side, one angle is broader and more obtuse producing a less regular triangular form, dorsal ridge less sharp, more rounded, glabrous but shortly hairy at the hilum, apex broadly rounded, seed width broader, color uniformly dull brown to grey-brown (Fig. 1S).

#### Phylogenetic position.

Morphological analysis shows that the new species is superficially similar to *Sida angustifolia*. Molecular phylogenetic analyses based on Bayesian inference and maximum likelihood recovered *Sida gathwalarum* as a distinct and well-resolved lineage within the genus *Sida*. This new species forms a closely allied clade with *Sida acuta*, indicating a recent common ancestry; however, it is clearly separated from *S. acuta* by a distinct branch, reflecting measurable genetic divergence. The clade comprising *S. gathwalarum* is supported by high posterior probability (100) and bootstrap value (100), reinforcing the robustness of its phylogenetic placement. Importantly, *S. gathwalarum* does not collapse into *S. acuta* or any other congeneric species but instead maintains an independent evolutionary lineage within the inferred topology. This molecular distinction is congruent with the observed morphological differences, thereby providing integrative evidence for its recognition as a separate species. Consequently, both molecular and morphological evidence strongly support the taxonomic delimitation of *Sida gathwalarum* as a new and independent species within *Sida* sect. *Sidae* (Fig. 3).

**Figure 2.**
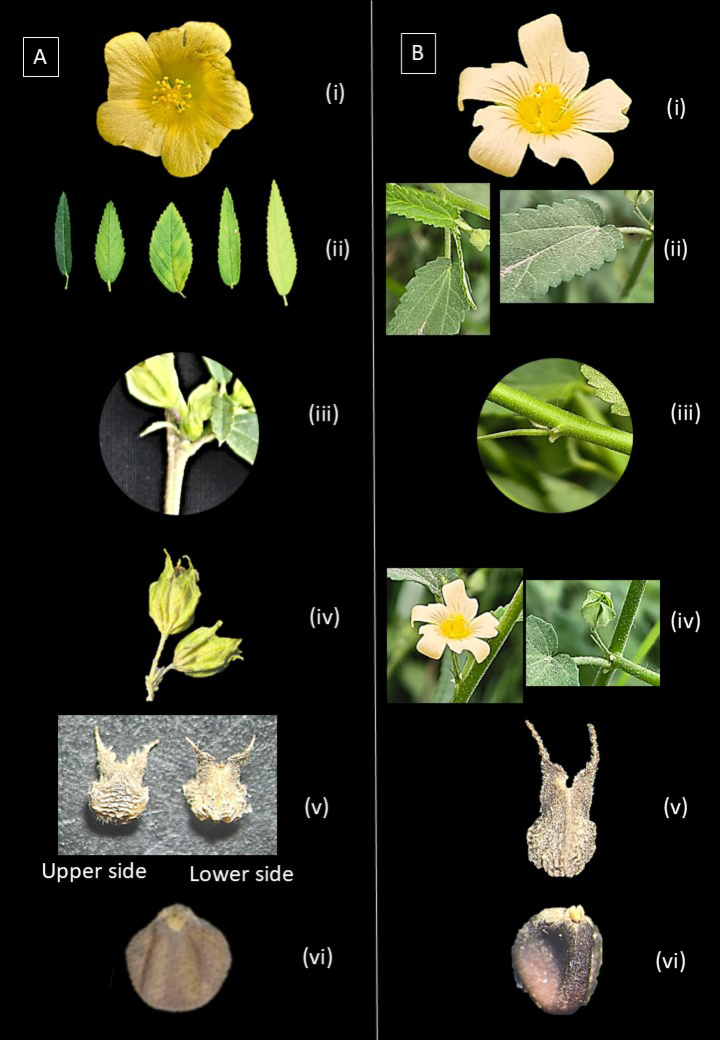
Comparative morphological features of *Sida gathwalarum* (**A**) and *Sida angustifolia* (**B**). **i**. Petal shape; **ii**. Leaf base; **iii**. Spiny emergences (spurs) at the base; **iv**. Articulation between peduncle and pedicel; **v**. Mericarp shape; **vi**. Seed shape.

**Figure 3.**
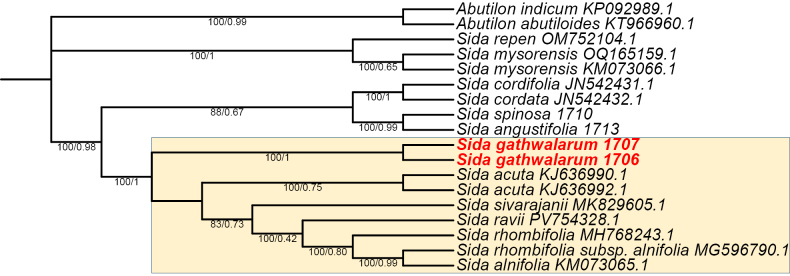
Maximum likelihood tree from analysis of nrDNA (ITS) of 14 species of *Sida*. The bootstrap values (BS) of ML and posterior probabilities (PP) of BI are listed at each node (PP/BS). The new species is highlighted in red. The yellow-shaded box indicates species belonging to *Sida* sect. *Sidae*.

**Figure 4.**
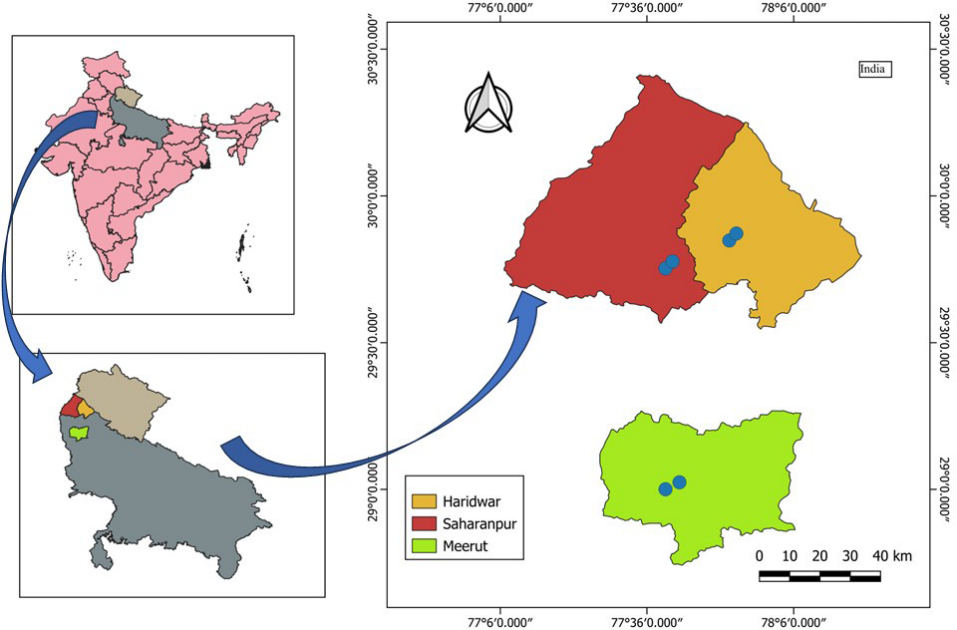
Distribution of *Sida gathwalarum*. Known distribution of the species in northern India based on five collections from Meerut (Uttar Pradesh) and Roorkee, Haridwar (Uttarakhand) (blue circle; map created with QGIS).

#### Phenology.

Flowering and fruiting occur almost throughout the year. The flowers open at 11:00 a.m. during summer and after 12:00 noon during winter.

#### Etymology.

The specific epithet gathwalarum honors the Gathwala (Gathwal) Khap, a traditional North Indian community to which the discoverers of this species belong.

#### Distribution and habitat.

*Sida gathwalarum* is currently known from several locations in Meerut and Saharanpur, Uttar Pradesh, and Roorkee, Haridwar (Uttarakhand), India (Fig. 3). It grows along open roadsides and wastelands, occurring sympatrically with *Sida acuta* Burm.f. and *Sida rhombifolia* subsp. *retusa* (L.) Borss. Waalk. Other associates of this species are *Achyranthes aspera* L., *Triumfetta rhomboidea* Jacq., *Urena lobata* L., *Setaria verticillata* (L.) P. Beauv., *Cynodon dactylon* (L.) Pers., *Digitaria ciliaris* (Retz.) Koeler, and *Oplismenus burmannii* (Retz.) P. Beauv.

#### Systematic position.

Based on molecular phylogenetic analyses, the new species is inferred to be closely allied to *Sida acuta*, which is a member of *Sida* sect. *Sidae* (Sivarajan and Pradeep 1996). Accordingly, the new species is also placed within *Sida* sect. *Sidae*.

#### Additional specimens examined (paratypes).

India • Uttar Pradesh: Saharanpur, Shahpur, 29.758°N, 77.657°E, 10 December 2025, Inam Malik & Vijai Malik 1707 (WII!); • Uttar Pradesh, Saharanpur, Badoli, 29.917°N, 77.656°E, 12 December 2025, Inam Malik & Vijai Malik 1708 (WII!).

#### Conservation status.

*Sida gathwalarum* is currently documented from a few scattered localities in the Meerut and Saharanpur districts of Uttar Pradesh and Roorkee in the Haridwar district of Uttarakhand, India. The habitats of this species are highly susceptible to anthropogenic disturbances, particularly due to urbanization and developmental works. The species appears to be underreported rather than genuinely rare. It commonly occurs along open roadsides and in wastelands. Based on its apparent distribution and habitat preference, the species is recommended for assignment to the IUCN Red List category Least Concern (LC) (IUCN Standards and Petitions Committee 2024).

##### Key to genus *Sida* in India (modified from Santosh Kumar et al. 2021 and Sivarajan and Pradeep 1996)

**Table:** 

1	Plant prostrate	***S. cordata***
–	Plant erect	**2**
2	Leaves palminerved	**3**
–	Leaves penninerved	**4**
3	Plants usually viscid, aromatic, staminal column glabrous	***S. mysorensis***
–	Plants not viscid, non-aromatic, staminal column pubescent	***S. elongata* var. *balica***
4	Petioles with 1–3 spiny emergences (spurs) at base	**5**
–	Petioles without spiny emergence (spur) at base	**6**
5	Peduncle long (≥ 0.5 cm); mericarps without a distinct notch	***S. spinosa***
–	Peduncle short (≤ 0.5 cm); mericarps with a distinct notch	***S. angustifolia***
6	Stipules dissimilar in shape	**7**
–	Stipules similar in shape	**8**
7	Leaves not distichous, mericarps dissimilar in shape	***S. gathwalarum***
–	Leaves distichous, mericarps similar in shape	***S. acuta***
8	Leaf margin entire	***S. linifolia***
–	Leaf margin not entire	**9**
9	Mericarp indehiscent	**10**
–	Mericarp dehiscent at apex	**11**
10	Fruiting pedicels 2.0–3.0 cm long	***S. ovata***
–	Fruiting pedicels 0.3–0.7 cm long	***S. rhomboidea***
11	Pedicels non-articulate	**12**
–	Pedicels articulate	**14**
12	Leaves concolorous	***S. scabrida***
–	Leaves not concolorous	**13**
13	Apex of mericarp beaked with a single muticous process, glabrous	***S. rhombifolia***
–	Apex of mericarp with a pair of linear divergent awns, stellately hairy	***S. keralensis***
14	Leaves concolorous	***S. pradeepiana***
–	Leaves not concolorous	**15**
15	Leaves near to the base of the stem always obovate, mericarp with a pair of short, stellately hairy mucronate at apex	***S. alnifolia***
–	Leaves otherwise, mericarp with a pair of short divergent glabrous awns	***S. subcordata***

## Supplementary Material

XML Treatment for Sida gathwalarum

## Additional information

### Artificial Intelligence (AI) use

The authors accept full responsibility for the content of the manuscript, including the disclosure of any use of AI.

No AI tools were used in the preparation of this manuscript.

### Author ORCIDs

S. Malik https://orcid.org/0009-0004-2507-9365

I. Malik https://orcid.org/0000-0002-9714-0661

V. Malik https://orcid.org/0000-0001-6991-7755

### Data availability

All of the data that support the findings of this study are available in the main text.
